# Human iPS Cells Loaded with MnO_2_-Based Nanoprobes for Photodynamic and Simultaneous Enhanced Immunotherapy Against Cancer

**DOI:** 10.1007/s40820-020-00452-y

**Published:** 2020-06-16

**Authors:** Yanlei Liu, Jingxing Yang, Bin Liu, Wen Cao, Jingpu Zhang, Yuming Yang, Lijun Ma, Jesus Martinez de la Fuente, Jie Song, Jian Ni, Chunfu Zhang, Daxiang Cui

**Affiliations:** 1 Institute of Nano Biomedicine and Engineering, Key Laboratory for Thin Film and Microfabrication of Ministration of Education, Shanghai Engineering Research Centre for Intelligent Diagnosis and Treatment Instrument, Department of Instrument Science and Engineering, School of Electronic Information and Electrical Engineering, Shanghai Jiao Tong University, 800 Dongchuan Road, Shanghai, 200240 People’s Republic of China; 2 National Engineering Center for Nanotechnology, Shanghai, 200240 People’s Republic of China; 3 Department of Nuclear Medicine, Rui Jin Hospital, School of Biomedical Engineering, Shanghai Jiao Tong University, Shanghai, 200240 People’s Republic of China; 4 Scientific Research Center, Shanghai Public Health Clinical Center, Fudan University, Shanghai, 201508 People’s Republic of China; 5 Tongren Hospital, Shanghai Jiao Tong University School of Medicine, 1111 XianXia Road, Shanghai, 200336 People’s Republic of China

**Keywords:** Human iPS, MnO_2_@Ce6 nanoprobes, Photodynamic therapy, Immunotherapy, Cancer

## Abstract

**Highlights:**

- MnO_2_@Ce6 nanoprobes-loaded-iPS cells (iPS-MnO_2_@Ce6) were developed for enhanced photodynamic and immunotherapy against cancer.
- Under the guidance of multi-mode real-time imaging, iPS-MnO_2_@Ce6 achieved an enhanced photodynamic therapeutic effect and stimulated a strong anti-tumor immune response in the tumor-bearing mouse.

**Abstract:**

How to trigger strong anti-tumor immune responses has become a focus for tumor therapy. Here, we report the human-induced pluripotent stem cells (iPSs) to deliver MnO_2_@Ce6 nanoprobes into tumors for simultaneous photodynamic therapy (PDT) and enhanced immunotherapy. Ce6 photosensitizer was attached on manganese dioxide (MnO_2_) nanoparticles, and resultant MnO_2_@Ce6 nanoprobes were delivered into mitomycin-treated iPSs to form iPS-MnO_2_@Ce6 nanoprobes. The iPS-MnO_2_@Ce6 actively targeted in vivo tumors, the acidic microenvironment triggered interaction between MnO_2_ and H_2_O_2_, released large quantities of oxygen, alleviated hypoxia in tumor. Upon PDT, singlet oxygen formed, broken iPSs released tumor-shared antigens, which evoked an intensive innate and adaptive immune response against the tumor, improving dendritic cells matured, effector T cells, and natural killer cells were activated. Meanwhile, regulatory T cells were reduced, and then the immune response induced by iPS-MnO_2_@Ce6 was markedly stronger than the immune reaction induced by MnO_2_@Ce6 (*P* < 0.05). The iPS-MnO_2_@Ce6 markedly inhibited tumor growth and metastasis and reduced mortality in mice models with tumor. Human iPSs loaded with MnO_2_-based nanoprobes are a promising strategy for simultaneous PDT and enhanced immunotherapy against tumor and own clinical translational prospect.
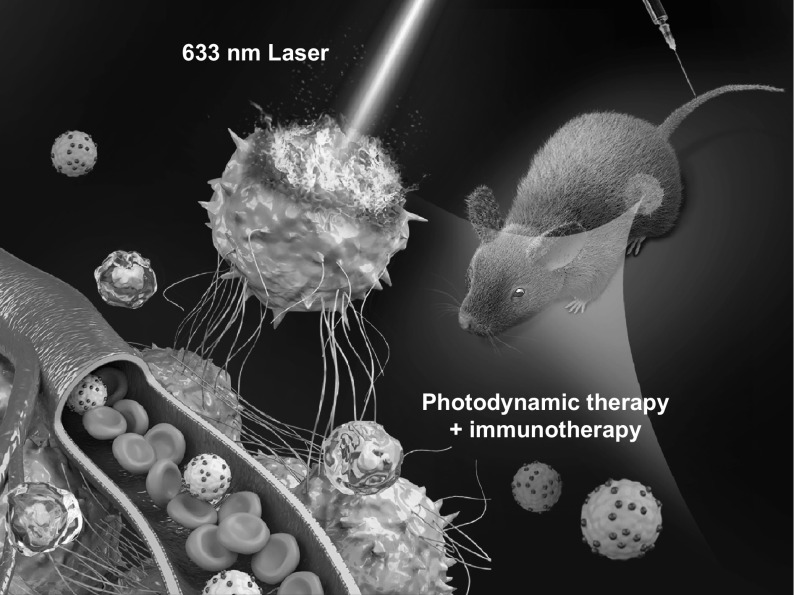

**Electronic supplementary material:**

The online version of this article (10.1007/s40820-020-00452-y) contains supplementary material, which is available to authorized users.

## Introduction

The occurrence and progression of tumors is highly correlated with tumor cell proliferation and evasion of the immune surveillance system [1–3]. Recently, studies on photodynamic therapy (PDT) and its potential anti-tumor immune responses have profoundly elucidated the complex interaction between tumor cells and the immune system [4–8]. Therefore, new therapeutic strategies aim to remove tumor cells and/or trigger the immune system to destroy the tumor [9–11]. Several anti-tumor immunotherapy strategies have been reported recently. In particular, anti-CTLA-4 and PD-L1/PD1 antibodies have been successfully used for anti-tumor immunotherapy by blocking the immune escape pathway adopted by tumors [12–14]. A systematic anti-tumor effect has been reported by combining photodynamic therapy with immune checkpoint inhibitors—proteins that activate immune T cells, allowing them to kill cancer cells [15–17]. PDT, in particular, is unique because unlike radio- and chemo-therapy, which suppress the immune system, PDT kills malignant cells and stimulates the host immune system to invade the tumor. The problem that lies in the photosensitizers used in PDT is that they have poor photo- and thermal stability [18, 19], rapidly clearing from the blood [20, 21], not accumulating sufficiently in the targeted tumor and low singlet oxygen production due to the hypoxic tumor microenvironment [22, 23]. These drawbacks inevitably cause inefficient therapeutic effects that might stop the body to trigger robust immune responses so as to completely eradicate the remaining tumor cells.

Embryonic stem cells (ESCs)—pluripotent stem cells derived from embryos—share the same transcriptome spectra and antigens with tumor cells [24, 25] and have great potential to be used as anti-tumor immunotherapy vaccines [26]. However, ethical constraints have limited their use. One promising alternative to ESCs is induced pluripotent stem cells (iPSs)—cells derived from the patient’s own tissues that have been reprogrammed into a pluripotent stem cell [27, 28] and thus completely overcome the ethical constraints [29–31]. iPSs share nearly identical gene expression and surface markers with ESCs [32, 33] and therefore have similar cellular and molecular properties to cancer cells. In our previous study, human iPS cells were used to realize targeted delivery of gold nanorods and PDT effect, the results showed that iPSs were a good delivery system [34]. We also confirmed that mitomycin-treated iPS cells exhibited good biocompatibility and kept the tumor-targeted ability [35]. The prepared biodegradable CaCO_3_/MnO_2_/PD-1 siRNA nanocomplexes exhibited enhanced PDT and PD-L1 immunotherapy [36]. It is observed that MnO_2_@Ce6 nanoparticles may modulate the tumor microenvironment by producing enough oxygen to overcome the key bottleneck of the hypoxia in tumor [37].

**Scheme 1:**
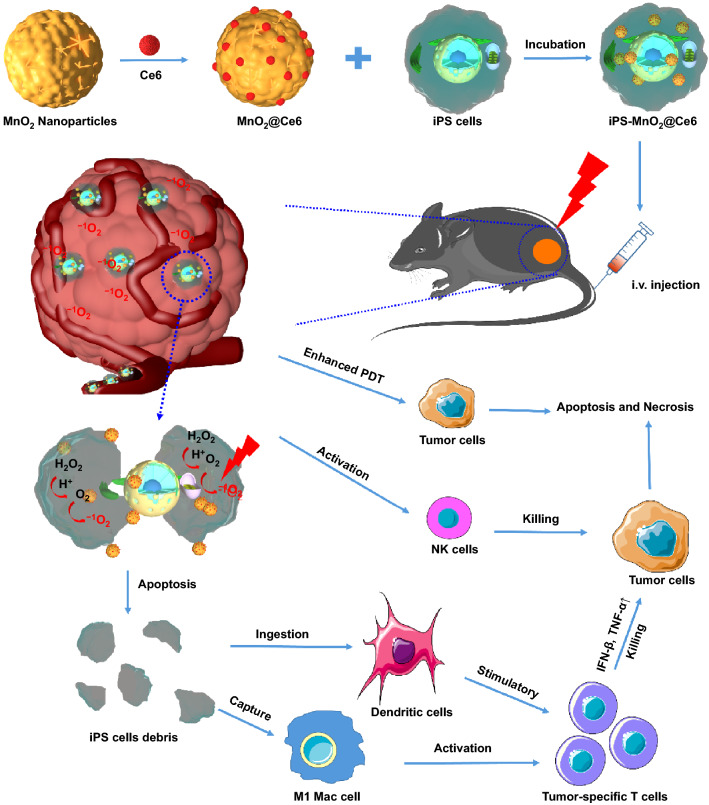
Schematic representation of iPS-MnO_2_@Ce6-mediated photodynamic therapy and anti-tumor immune responses for combinational cancer therapy

Herein, we report for the first time mitomycin-treated iPSs to deliver MnO_2_@Ce6 nanoprobes into tumors for simultaneous photodynamic and enhanced immunotherapy (Scheme 1). The iPSs served as both a delivery vector for MnO_2_@Ce6 and a source of tumor immune stimulating antigens after laser irradiation during PDT. The results showed that the MnO_2_@Ce6-loaded iPSs actively targeted tumors in vivo and caused the infiltration of multiple types of immune cells that mounted an effective anti-tumor immune response. More important, we also observed that MnO_2_@Ce6 could induce anti-tumor immunoreaction in C57 mice models loaded with lung cancer. Treated animals showed inhibited tumor growth and lower mortality than those in control groups. This work suggests that the iPS loaded with MnO_2_@Ce6 nanoprobes have great potential in the synergistic anti-tumor therapy based on photodynamic/immunotherapy strategies.

## Experimental Section

### Materials

Potassium permanganate and polycyclic-aromatic hydrocarbons (PAH, 15000) were obtained from Sinopharm Chemical Reagent Co., Ltd. (Shanghai, China). Indocyanine green (Ce6) was purchased from Sigma-Aldrich (St. Louis, USA). Lewis cells were available in Cell Bank of Type Culture Collection of Chinese Academy of Sciences. Deionized (DI) water with resistivity of 18.2 MΩ was used in all of the experiments.

### Preparation of Nanoprobes

MnO_2_ nanoparticles were synthesized according to the previously reported preparation method with some modifications [38]. In brief, 10 μL of 11.8 mg mL^−1^ potassium permanganate solution was added to 12 mL of 4.1 μg mL^−1^ PAH aqueous solution, and then the mixture was ultrasound dispersed for 30 s. Then, the mixture was placed at room temperature without any interruption. After about 5 min of reaction time, the light purple reaction liquid turned into light brown yellow, which indicated the formation of MnO_2_ nanoparticles. The as-prepared MnO_2_ nanoparticles were stored at 4 °C for further use. Finally, the nanoprobe (MnO_2_@Ce6) was obtained through simply stirring a mixture of MnO_2_ and Ce6 for 12 h at 4 °C in the dark, and the product was washed two times with DI water to obtain the purified MnO_2_@Ce6.

### Characterization of Nanoprobes

The morphology of MnO_2_@Ce6 was characterized by transmission electron microscopy (TEM) and scanning electron microscopy (SEM), respectively. Dynamic light scattering and zeta potential of these nanoprobes were measured using a NICOMP 380 ZLS Zeta Potential/Particle sizer. The UV/vis absorption spectra of Ce6, MnO_2_ nanoparticles, and MnO_2_@Ce6 were recorded on a Varian Cary 50 spectrophotometer. The fluorescence spectra of Ce6 and MnO_2_@Ce6 were measured on a Hitachi F-4600 spectrophotometer. The percentages of Ce6 release from MnO_2_@Ce6 were measured at 37 °C in the PBS with pH of 5.0, 6.0, and 7.4, respectively. The amount of released CE6 was measured on a UV–vis spectrophotometer.

### Singlet Oxygen Detection

Singlet oxygen sensor green (SOSG) was usually employed to detect the generation of ^1^O_2_, which was determined by measuring recovered SOSG fluorescence (excitation = 494 nm). In our experiment, the generation of ^1^O_2_ from Ce6 and MnO_2_@Ce6 (equivalent Ce6) was measured by SOSG under the concentration of 2.5 × 10^6^ M.

### Cell Culture

The human iPS were purchased from Nuwacell Co., Ltd (China, Anhui) and cultured at 37 °C in a humidified incubator containing 5% CO_2_. In this study, the iPS cells culture and mitomycin treatment were performed as we previously reported [35].

### Cellular Uptake

iPS cells were seeded onto a 35 mm borosilicate chambered covered glass at a density of 5 × 10^4^ cells mL^−1^. After 72-hours’ incubation, the medium was replaced with fresh medium and incubated with Ce6 or MnO_2_@Ce6 in the dark for 12 h. Fluorescence images of cellular were acquired by a confocal fluorescence microscope ((Lecia SP8 STED 3X) with an excitation wavelength of 633 nm. Besides, one part of the cells treated with MnO_2_@Ce6 was collected following standard methods for TEM cell sample preparation. Finally, the distribution and degradation of nanoprobes in iPS cell was observed by TEM.

### Quantification of MnO_2_@Ce6–Loaded iPS Cells

To measure the uptake amount of MnO_2_@Ce6 by iPS cells, the iPS cells were incubated with MnO_2_@Ce6 for 24 h. At the predetermined time points- 1, 4, 8, 14, and 24 h, the iPS cells were washed three times with PBS to remove unloaded MnO_2_@Ce6 and then were diluted into 1 mL PBS with 2% HNO_3_, and the Mn ions concentrations in these cells were determined by inductively coupled plasma-mass spectrometry (ICP-MS).

### Cell Viability

The effect of MnO_2_@Ce6 on iPS cells’ proliferation and viability was monitored using a real-time cell analyzer (RTCA Analyzer, Roche). The iPS cells were plated at a density of 1 × 10^4^ cells/well on 96-well plate. After 24 h incubation, the medium was replaced with fresh medium containing of PBS, Ce6 or MnO_2_@Ce6, respectively. Based on the electronic impedance across microelectrodes integrated into the bottom of cell culture, E-Plates was captured every 5 min during 60 h period. Finally, the curves of time-normalized cell index were plotted by RTCA software.

### Measurement of Intracellular Oxygen Generation

The changes in intracellular oxygen level (O_2_ %) in MnO_2_@Ce6-loaded iPS cells were measured by MitoXpress Intra kit. In briefly, iPS cells were planted on 12-well plate and then incubated with PBS, MnO_2_, and MnO_2_@Ce6, and then the changes of O_2_ % in the iPS cells were conducted according to the standard procedure of the kit, followed by confocal fluorescence imaging.

### Measurement of Intracellular ROS Generation

In order to measure the amount of intracellular ROS under the NIR laser irradiation, DCF-DA was selected to monitor ROS generated by MnO_2_@Ce6 in iPS cells. iPS cells were seeded on 12-well and then incubated with PBS, Ce6, and MnO_2_@Ce6. After 8 h of incubation, the medium was replaced with fresh medium containing DCF-DA (10 nM) for the further incubation of 20 min in the dark, followed by 633 nm laser irradiation (6 min, 0.5 W cm^−2^), and finally the cells were collected and measured by flow cytometry.

### In Vitro Photodynamic Effect of MnO_2_@Ce6 on iPS Cells

iPS cells were incubated with PBS, Ce6, and MnO_2_@Ce6 for 8 h and then were carefully rinsed three times with PBS to remove the unloaded nanoprobes. After that, these nanoprobes-loaded iPS cells were irradiated by the 633 nm laser with a power of 0.5 W cm^−2^ for 6 min. After laser treatment, the treated iPS cells were identified by Calcein AM and PI staining on confocal fluorescence microscopy.

### Animal and Tumor Model

Forty female BALB/c nude and 40 C57 mice, 4–6 weeks of age and weighting 18–22 g, were purchased from shanghai LAC laboratory animal Co., Ltd., and housed in an SPF grade animal center. The use of all mice/mouse in this study complied with the current ethical considerations: Approval of institutional Animal Care and Use Committee of Shanghai Jiao Tong University. The mice were anesthetized by isoflurane and 2 × 10^6^ Lewis cells suspended in 100 μL saline were transplanted into the mice subcutaneous.

### Photoacoustic Imaging

BALB/c nude mice with Lewis tumors were injected by a lateral tail vein with MnO_2_@Ce6 and iPS-MnO_2_@Ce6, and then, the real-time photoacoustic images and oxygen saturation (O_2_) of the tumor were recorded at various time points after i.v. injection with a Vevo LAZR Photoacoustic Imaging System. The tumor oxygen saturation was measured at two excitation wavelengths (750 and 850 nm), and all the photoacoustic images were recorded with the same parameter settings.

### Immunohistochemistry

Tumor-bearing C57 mice were i.v. injected with saline, MnO_2_@Ce6, and iPS-MnO_2_@Ce6. At the predetermined time, tumors were harvested 60 min after the intraperitoneal injection with pimonidazole hydrochloride (60 mg kg^−1^) (Hypoxyprobe-1 plus kit, Hypoxyprobe Inc). The remaining steps were performed according to the kit instructions. The images were captured by confocal microscopy (Leica SP5).

### In Vivo Fluorescence Imaging

A Bruker In Vivo FPRO imaging system was used to make real-time observation of the distribution and metabolism of MnO_2_@Ce6 loaded iPS cells in tumor-bearing BALB/c nude mice. When the tumor size reached ≈ 100 mm^3^, the tumor-bearing mice were injected by a lateral tail vein with 100 μL MnO_2_@Ce6 loaded iPS cells (Ce6 equivalent 10 mg kg^−1^) suspension, the NIR fluorescence images of Ce6 (excitation: 710 nm; integration time: 30 s) were obtained according to different injection times (0, 1, 6, 12, and 24 h) with the same parameter settings.

### In Vivo T1-MR Imaging

For the in vivo MR imaging, the tumor-bearing BALB/c nude mice were intravenously injected with 100 μL of saline containing the iPS-MnO_2_@Ce6 (50 μg Mn per mouse, *n* = 5). The T1-weight MR scanning images were obtained from a 3.0 T clinical MR imaging instrument (SOMATON Definition Flash, Siemens, Erlangen, Germany) at different injection time points (0, 1, 6, 12, and 24 h). All MR scanning images were performed with the same parameter settings.

### Flow Cytometry Analysis of Immune Cells

To systematically investigate immune cells involved in the anti-tumor immune responses, these tumors from C57 mice were collected and digested using collagenase, hyaluronidase, and DNase to produce single-cell suspensions. The collected single cells were first incubated with FcRblock and stained with several fluorochrome-conjugated antibodies: CD3-APC (Biolegend, Clone: 17A2), CD45-APC/Fire750 (Biolegend, Clone: 30-F11), CD8a-PerCP/Cyanine5.5 (Biolegend, Clone: 53-6.7), CD4-FITC (Biolegend, Clone: RM4-5), CD45-APC/Fire750 (Biolegend, Clone: 30-F11), I-A/I-E-PerCP/Cyanine5.5 (Biolegend, Clone: M5/114.15.2), CD11c-APC (Bioscience, Clone: N418), CD80-PE/Cy7 (Bioscience, Clone: 16-10A1), CD86-PE (eBioscience, Clone: GL-1), FOXP3-PE (Bioscience, Clone: MF-14), CD25-Brilliant Violet 421™ (Bioscience, Clone: PC61), F4/80-APC (Bioscience, Clone: BM8), CD16/32-PE/Cy7 (Bioscience, Clone: 93), CD206-PE (Bioscience, Clone: C068C2), CD11b-FITC (Bioscience, Clone: M1/70), CD19-FITC (Bioscience, Clone: 1D3/CD19), CD49b-PE/Cy7 (Bioscience, Clone: HMα2) and then analyzed using FCM. Antibodies were diluted 100 times in the study. The total number of tumor cells (1 × 10^5^) was collected for analyzing specific immune cells in various treatment groups. Correspondingly, the flow gating strategies are shown in detail in Fig. S15. For analysis of CD8+ CTL cells or CD4+ T helper cells, the FCM gate strategy was defined as Live CD45+ CD3+ CD8+ CD4− and Live CD45+ CD3+ CD8− CD4+, respectively. Mature DCs were gated on Live CD45+ CD11c+ I-A/I-E+ CD80+ CD86+, B/NK cells were gated on Live CD45+ CD3− CD19+/CD49+, and macrophages polarization was gated on Live CD45+ CD11b+ F4/80+ CD16/32 (M1 type) or CD206+ (M2 type).

### In Vivo Therapy

Lewis lung tumor xenografts were implanted subcutaneously into C57 mice by injecting Lewis cells into the right hind limb. When tumor size reached 100 mm^3^, the mice were treated with an intravenous injection of saline, free Ce6, MnO_2_@Ce6-loaded iPS cells (Ce6 equivalent 10 mg kg^−1^). Up to 24 h after i.v. injection, the tumor region was irradiated using a 633 nm laser at a power of 0.5 W cm^−2^ with 1 min interval for every 3 min of irradiation. After the last irradiation treatment, tumor size was measured every three days by digital vernier caliper. Meanwhile, the mice injected with the same nanoplatform as above-mentioned were not exposed to lasers as control groups. Sections of the tumor tissue 14 days after i.v. injection from each group were used for TUNEL and Ki67 staining and H&E staining according to the manufacture's instructions.

### Statistical Analysis

All experiments were performed in triplicate unless otherwise indicated. These data were expressed as mean ± standard deviation. Statistical significance was determined using a two-tailed student’s *t* test. A probability level of 95% (*P* < 0.05) was considered significantly different.

## Results and Discussion

**Fig. 1:**
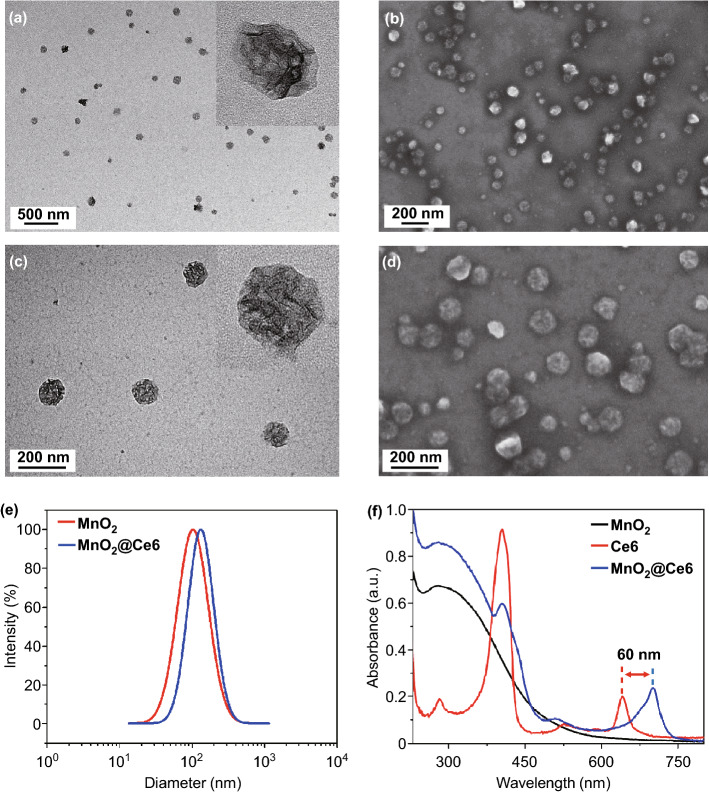
Characterization of MnO_2_@Ce6. **a** TEM and **b** SEM image of MnO_2_ nanoparticles. **c** TEM and **d** SEM image of MnO_2_@Ce6 nanoprobes. **e** Size distribution of MnO_2_ and MnO_2_@Ce6. **f** UV–vis absorption spectra of MnO_2_, Ce6, and MnO_2_@Ce6 solutions

We synthesized MnO_2_ nanoparticles according to a modified reduction method. Briefly, potassium permanganate solution was mixed with polycyclic-aromatic hydrocarbon aqueous solution (PAH) and dispersed by ultrasound to form PAH-modified MnO_2_ nanoparticles. Stirring PAH-modified MnO_2_ nanoparticles with photosensitizer, Ce6, formed the MnO_2_@Ce6 nanoprobe. TEM images revealed that the MnO_2_ nanoparticles were spherical with walnut-like wrinkles on the surface (Fig. 1a), while SEM images showed they had an average diameter of around 100 nm and were uniform in size and well-dispersed (Fig. 1b). The surface wrinkles increased the nanoparticle surface area (allowing more Ce6 to be loaded) and protected Ce6 from light-/thermal-induced degradation (Fig. 1c, d). Because PAH has a large amount of amide on the surface, the PAH-modified MnO_2_ nanoparticles showed a positive zeta potential of 30 ± 3.03 mV, while MnO_2_@Ce6 exhibited a less positive zeta potential of 10 ± 1.25 mV (see Fig. S1). Dynamic light scattering (DLS) further confirmed the MnO_2_ and MnO_2_@Ce6 nanoparticles have a narrow size distribution with an average diameter of 100 ± 9.5 and 108 ± 7.5 nm, respectively (Fig. 1e). From the UV–Vis absorption spectra, the Q(I) band peak of Ce6 after loading onto MnO_2_ (blue curve in Fig. 1f) was seen to red-shift by 60 nm to 700 nm, suggesting that Ce6 was successfully loaded onto MnO_2_ in an aggregated state, which is consistent with previous reports. The loading content of Ce6 was estimated to be 9.1% by UV–vis spectroscopy. As shown in Fig. S2, the fluorescence quenching of loaded Ce6 was confirmed by fluorescence spectra of MnO_2_@Ce6, which verifies that the loaded Ce6 is in a stable aggregation state.

At pH 7.4, TEM showed MnO_2_@Ce6 did not experience any significant changes in morphology (see Fig. S3). However, as pH decreased from 7.4 to 3.0, degradation increased gradually, demonstrating the nanoprobes are highly sensitive to acidic environments. Next, we investigated the pH-triggered release performance of the loaded Ce6 from MnO_2_@Ce6 in varying pH (5.0, 6.5, and 7.4) environments. As shown in Fig S4, the release files of Ce6 confirm the PH-responsive release characteristics of the nanoprobe. It is known that MnO_2_ is highly reactive toward H_2_O_2_, producing O_2_, and Mn^2+^ by consuming H^+^ ions [39]. To test the reactivity of our nanoprobes, we incubated different concentrations of MnO_2_@Ce6 with H_2_O_2_ in saline at pH 6.5 for 200 s and continuously measured the O_2_ levels. No changes in O_2_ levels were observed for the control group without nanoprobes. In groups treated with 40 and 60 μg of MnO_2_, O_2_ level increased rapidly in the first 100 s before reaching a peak of 28 and 58 mg L^−1^, respectively (Fig. S5). One of the reasons why PDT is inefficient is the hypoxic environment in tumors, which lacks oxygen for photosensitive drugs to generate singlet oxygen (^1^O_2_). The efficient generation of O_2_ from the degradation of MnO_2_@Ce6 suggests that these nanoprobes could potentially be used to improve the production of ^1^O_2_ by photosensitizers during PDT. To evaluate this possibility, we measured the generation of ^1^O_2_ from free Ce6 and MnO_2_@Ce6 under a 633 nm laser irradiation. As expected, at the same concentration of Ce6, the amount of ^1^O_2_ produced by MnO_2_@Ce6 was nearly 1.3-fold higher than free Ce6 after 3 min of irradiation (Fig. S6). Furthermore, prolonged irradiation enhanced the accumulation of ^1^O_2_ (Fig. S7). These results show the enhanced production of ^1^O_2_ is due to MnO_2_, which protects Ce6 from thermal-/light-induced degradation and provides a source of oxygen for Ce6.

**Fig. 2:**
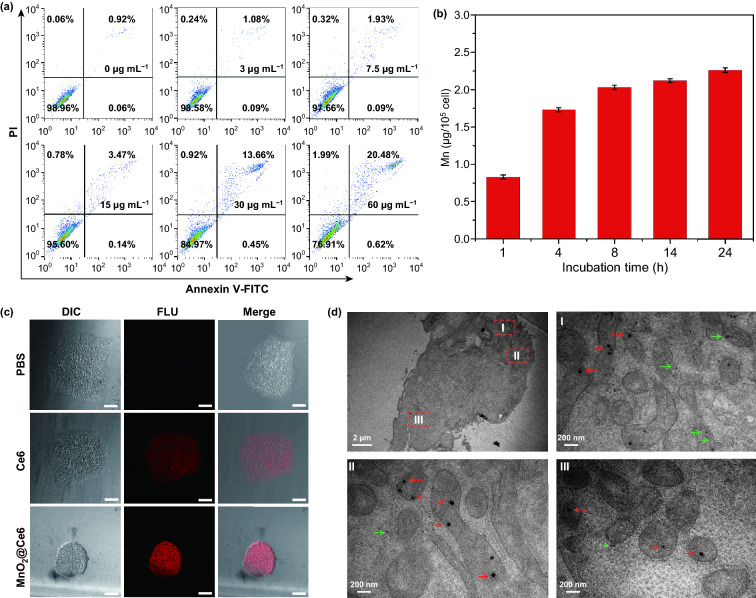
The loading of nanoprobes by iPS cells. **a** Flow cytometric analysis of iPS cells apoptosis induced by nanoprobes (Mn concentration: 0-60 μg mL^−1^). **b** Quantitative cellular uptake of MnO_2_@Ce6 by iPS cells measured by ICP-MS. **c** Confocal laser scanning microscopy images of iPS cells treated with Ce6 and MnO_2_@Ce6, all scale bars are 100 μm. **d** Representative TEM images of iPS cells incubated with nanoprobes. In the TEM images, the red arrows indicate the nanoprobe without degradation and the green indicate the nanoprobe with degradation

To determine the optimal concentration of MnO_2_@Ce6 nanoprobe to use, we incubated iPSs with different concentrations of the nanoprobe and analyzed the cytotoxic response by flow cytometry. As shown in Fig. 2a, no cytotoxicity was seen up to 30 μg mL^−1^, making it a suitable concentration for loading the nanoprobe into iPSs. Furthermore, at 30 μg mL^−1^ MnO_2_@Ce6, the treated group showed similar cell proliferation rates to the phosphate-buffered saline (PBS) control group as monitored by real-time amplification (RTCA), further proving this concentration of MnO_2_@Ce6 is safe for iPSs (Fig. S8). To determine the uptake of the nanoprobes, we incubated iPSs with 30 μg mL^−1^ MnO_2_@Ce6 at different times and quantified the uptake by ICP-MS. Nanoprobe uptake increased significantly in the first 4 h before slowing down and reaching a plateau at 8 h (Fig. 2b). From these experiments, to safely load MnO_2_@Ce6 into iPSs for all subsequent experiments, we incubated 30 μg mL^−1^ MnO_2_@Ce6 with iPSs (at 21 μg/10^6^ cells) for 8 h.

Confocal fluorescence microscopy images revealed iPSs carrying free Ce6 and MnO_2_@Ce6 showed prominent near-infrared (NIR) fluorescence signals from Ce6 (Fig. 2c). However, fluorescence signals from MnO_2_@Ce6 were much stronger and more localized due to efficient endocytosis. TEM images show MnO_2_@Ce6 were found in different organelles and the cytoplasm of the iPSs without any obvious specificity (Fig. 2d). However, it is worth noting that nanoprobes located in the cytoplasm and lysosomes were markedly degraded (red arrows in Fig. 2d), whereas those in the mitochondria retained its spheroidal shape (green arrows in Fig. 2d). This is likely due to the different pH values in these organelles; the exact mechanisms would need to be determined in the future.

Once inside the iPSs, the reaction between MnO_2_ and H_2_O_2_ leads to MnO_2_ degradation and O_2_ release, providing a source of O_2_ for the production of intracellular ^1^O_2_ under laser irradiation. To verify this, we used the oxygen-sensing probe, [(Ru(dpp)_3_)]Cl_2_, to measure O_2_ and flow cytometry to measure ^1^O_2_ inside iPSs containing the MnO_2_@Ce6 nanoprobe. Fluorescence images showed that the red fluorescence of [(Ru(dpp)_3_)]Cl_2_ decreased gradually over time and disappeared after 6 h, indicating the nanoprobe significantly increased the amount of O_2_ inside iPSs. After 6 min of laser irradiation, the intracellular concentration of ^1^O_2_ in nanoprobe-treated iPSs was significantly higher than Ce6-treated iPSs (Fig. S9). Together, these results demonstrate that the MnO_2_@Ce6 nanoprobes, which can facilitate the production of large quantities of ^1^O_2_ in the iPSs upon irradiation, are expected to produce an enhanced PDT effect when applied to cancerous tumors.

**Fig. 3:**
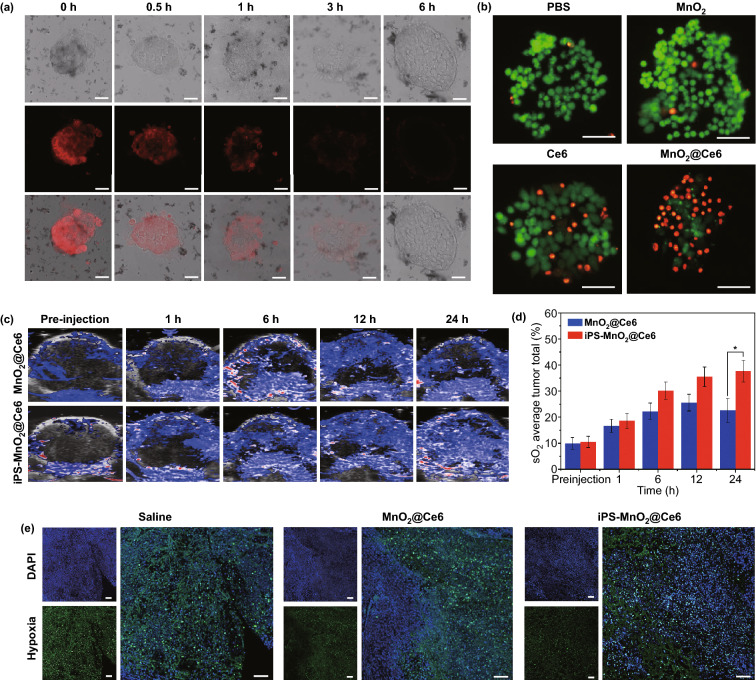
The excellent biological properties of iPS-MnO_2_@Ce6. **a** Confocal laser microscopy images of oxygen-sensing probe ([(Ru (dpp)_3_)]Cl_2_) in iPS cells showing the intracellular oxygen level incubated with nanoprobes for the indicated time. Scale bars are 50 μm. **b** Fluorescence images of Calcein AM (green)/PI(red) stained iPS cells incubated with free Ce6, MnO_2_, and nanoprobes. Scale bars are 50 μm. **c** Photoacoustic imaging showing saturated O_2_ (sO_2_) levels in tumors after injection of MnO_2_@Ce6 and iPS-MnO_2_@Ce6 at different time points. **d** The quantified tumor oxygen saturation levels calculated from **c**. Data were expressed as mean ± SD, (*n* = 3, *P < 0.05). **e** Representative immunofluorescence images of tumor sections after hypoxia staining. The nuclei and hypoxia regions were stained by DAPI (blue) and anti-pimonidazole antibody (green), respectively. Scale bars are 100 μm. (Color figure online)

Encouraged by the enhanced ^1^O_2_ generation capacity of the nanoprobes, we further examined their ability to kill iPSs through PDT using the LIVE/DEAD cell straining method. Almost no dead cells were observed in control groups treated with PBS and MnO_2_ (Fig. 3b). Nearly equal amounts of dying cells (stained red) and live cells (stained green) were observed in groups treated with free Ce6, suggesting that Ce6 alone cannot kill all cells. In contrast, nearly all iPSs were killed in the MnO_2_@Ce6-treated group, demonstrating that the enhanced PDT effect of MnO_2_@Ce6 is applicable in iPSs.

As mentioned above, iPSs carrying nanoparticles can migrate toward targeted tumors and improve the spatial distribution of the nanoparticle payload in tumor tissues. To explore whether the loaded MnO_2_@Ce6 would affect the ability of iPSs to target tumor tissues, the oxygen content in tumor tissues in mice was monitored in real-time by measuring vascular saturated O_2_ (sO_2_) at two excitation wavelengths (750 and 850 nm) using a photoacoustic scanner. The sO_2_ content in tumor tissues increased dramatically after injection with iPSs containing MnO_2_@Ce6 (iPS- MnO_2_@Ce6), reaching around 40% (4-fold higher than pre-injection) by 24 h post-injection. In the group treated with MnO_2_@Ce6 (without iPSs), sO_2_ content peaked at 12 h post-injection, which was just twofold higher than pre-injection and began to weaken thereafter. Consistent with the photoacoustic imaging results, immunofluorescence images of tumors showed that iPS-MnO_2_@Ce6 relieved tumor hypoxia (Fig. 3e). We believe iPSs had prolonged the retention of MnO_2_@Ce6 in tumor tissues, allowing O_2_ to be produced and accumulate inside the tumor.

**Fig. 4:**
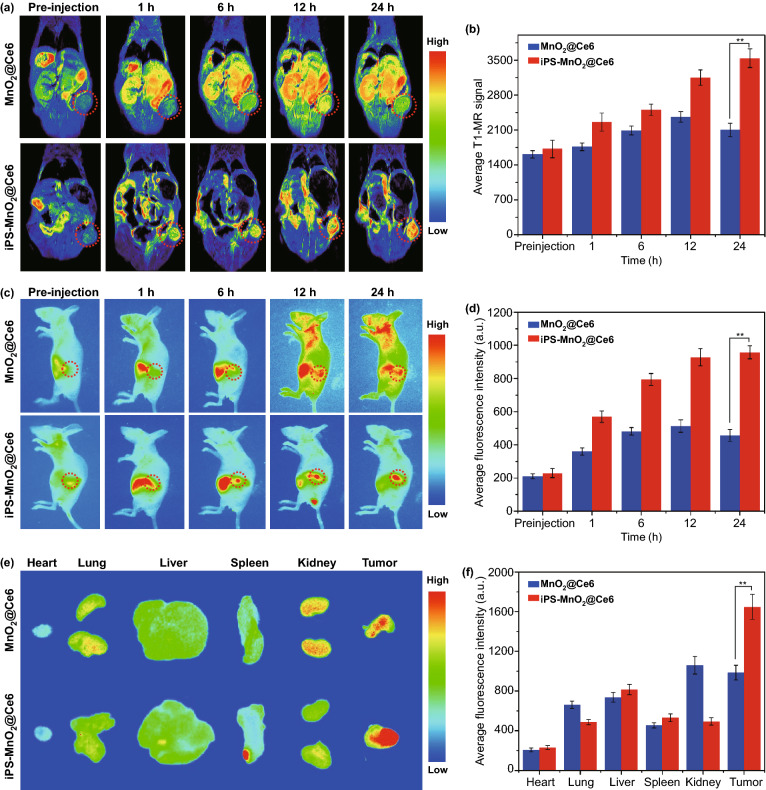
Distribution and metabolism of iPS-MnO_2_@Ce6 in tumor-bearing mice. **a** T1-MR images of tumor-bearing mice after the *i.v.* injection of MnO_2_@Ce6 and iPS-MnO_2_@Ce6 over a period of 24 h. **b** Quantitative analysis of T1-MR signal from tumor areas in (a). **c** Real-time fluorescence images of tumor-bearing mice after the *i.v.* injection of MnO_2_@Ce6 and iPS-MnO_2_@Ce6. **d** Quantitative analysis of fluorescence signal from tumor areas in **c**. **e** Ex vivo fluorescence images of major organs and tumor tissues. **f** Quantification of fluorescence signals from images shown in (e). Data were expressed as mean ± SD, (*n* = 3, **P < 0.001)

Using T1 magnetic resonance (T1-MR) and fluorescence imaging, we followed in real-time the distribution and metabolism of iPS-MnO_2_@Ce6 in tumor-bearing mice. Upon intravenous injection, the MnO_2_@Ce6-treated group showed strong MR signals in the liver, which increased in a stepwise fashion for the first 12 h before decreasing (Fig. 4a). In contrast, the MR signal observed 1 h post-injection in the iPS-MnO_2_@Ce6-treated group strengthened gradually during the imaging period. At its peak, the signal in the tumor treated with iPS-MnO_2_@Ce6 was nearly 1.5 times stronger than the MnO_2_@Ce6-treated group (Fig. 4b).

To examine the distribution of MnO_2_@Ce6 and iPS-MnO_2_@Ce6 in tumor-bearing mice, we measured the NIR fluorescence of Ce6 using real-time fluorescence imaging. Strong signals in the liver were observed in the MnO_2_@Ce6-treated groups 1 h post-injection (Fig. 4c, d). These signals decreased over time as Ce6 was gradually excreted from the liver. Of note is the very weak fluorescence seen in tumor tissues, indicating poor tumor accumulation of MnO_2_@Ce6. In the case of iPS-MnO_2_@Ce6-treated animals, fluorescence signals were detected in the tumor region within just 6 h after intravenous injection. The signals continued to increase thereafter and peaked at 24 h post-injection. Ex vivo images of major organs and tumor tissues further confirmed the high tumor targeting efficiency of iPS-MnO_2_@Ce6 (Fig. 4e, f). These results demonstrated that iPS-MnO_2_@Ce6 can effectively target tumor tissues and relieve hypoxia in the tumor microenvironment, resolving two key obstacles in PDT.

**Fig. 5:**
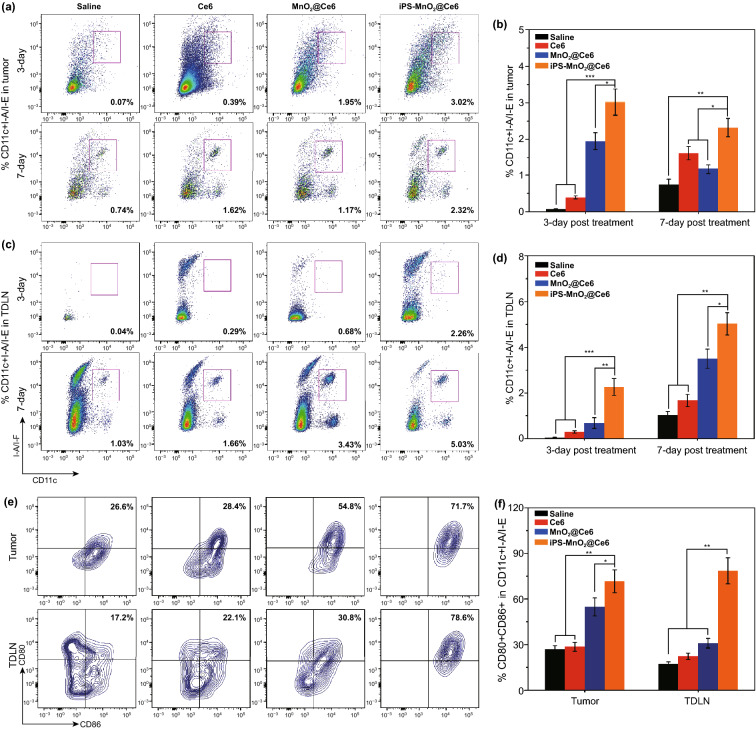
The infiltration and maturation of DCs treated by iPS-MnO_2_@Ce6-mediated photodynamic therapy in tumor tissues and lymph nodes. **a**–**d** DCs infiltrating in tumor tissues (**a**, **b**) and tumor-draining lymph nodes (TDLN) (**c**, **d**) were analyzed by flow cytometry after stained with Liver-Dead, CD45+, CD11c+, I-A/IE. **e, f** Mature DCs in tumor tissues and DTLN 7 days after the treatment were stained with CD80 and CD86 for flow cytometry assay. Data were expressed as mean ± SD, (*n* = 5, *P < 0.05, **P < 0.001, ***P < 0.001)

To investigate the involvement and response of immune cells stimulated by iPS-MnO_2_@Ce6 in laser-mediated PDT, we used flow cytometry to analyze the number of dendritic cells (DCs) that infiltrated the tumors and lymph nodes on day 3 and 7 after laser treatment. DCs are the main antigen-presenting cells that initiate, regulate and adapt the immune system. Upon exposure to antigens, DCs capture and process the antigens before entering a local tissue-draining lymph node, where they activate systematic tumor immune responses by presenting the major histocompatibility complex-peptides to T cells. On day 3 after laser treatment, flow cytometric analysis showed significant infiltration of CD11c+ I-A/I-E DCs in both tumors and lymph nodes of mice injected with iPS-MnO_2_@Ce6 (Fig. 5a, c). Compared to the saline-treated control group, approximately 3% and 2% more CD11c+ I-A/I-E DCs infiltrated, respectively, into the tumors and draining tumor lymph nodes (DTLN) of iPS-MnO_2_@Ce6-treated animals (Fig. 5b, d). On day 7 after treatment, the proportion of CD11c+ I-A/I-E DCs infiltrating the tumor and DTLN increased further. Moreover, the proportion of mature CD80+ CD86+ DCs in both the tumor and DTLN of animals treated with iPS-MnO_2_@Ce6 was significantly higher than those in the other treatment groups (Fig. 5e, f). Together, these results demonstrated that iPS-MnO_2_@Ce6 elicited an effective immune process of antigen presentation upon laser irradiation, simultaneously promoting PDT and immunotherapy.

**Fig. 6:**
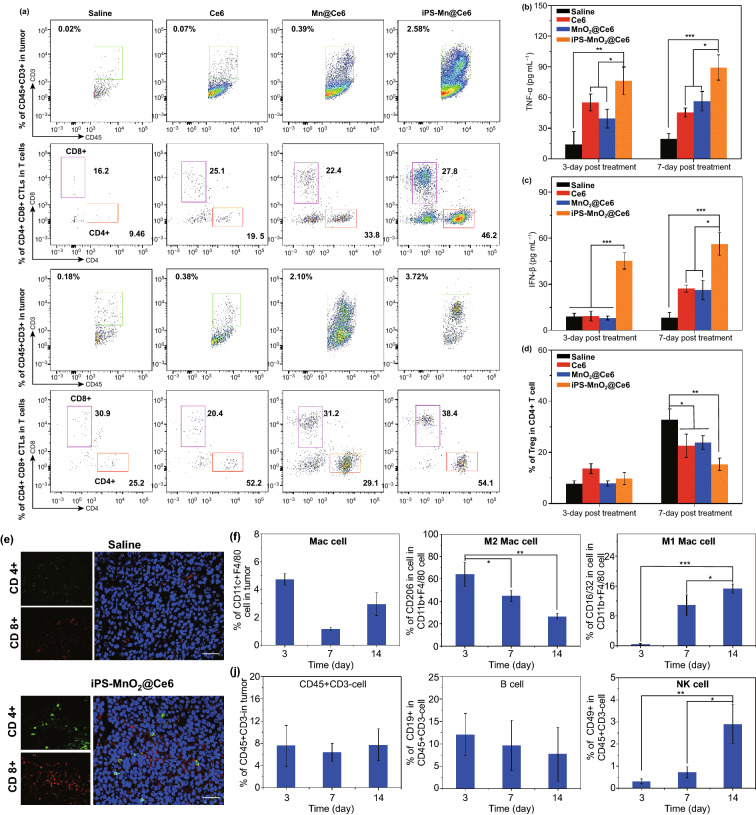
T cell response-based immunotherapeutic effect of iPS-MnO_2_@Ce6 in tumors. **a** Representative flow cytometric plots of CD4+ and CD8+ CTL in CD45+ CD3+ T cells for tumors from various groups at different time points post-laser-mediated treatments. The contents of **b** TNF-α and **c** IFN-β in tumor tissues from each groups at different time points after treatments. **d** The proportions of CD4+ Foxp3+ regulatory T cell (Treg) in CD4+ T cells. **e** Representative immunofluorescence images of tumor slides showing CD4+ and CD8+ T cells infiltrating into the tumor tissues. Green from CD4 antibody, red from CD8 antibody, and blue from DAPI. Scale bars are 50 μm. **f** The infiltrations of CD11c+ F4/80 cells (Mac cells), M2 Mac cell, and M1 Mac cell in tumors, **j** B cells and NK cells in tumors at different time points post treatments of iPS-MnO_2_@Ce6. Data were expressed as mean ± SD, (*n* = 5, *P < 0.05, ** *P *< 0.001, *** *P *< 0.001

To verify whether mature DCs successfully initiated T cell-mediated anti-tumor immune responses, we analyzed T cell profiles in tumor tissues by flow cytometry and immunofluorescence. After laser treatment for 3 or 7 days, it can be clearly seen from Fig. 6a that the proportion of CD45^+^CD3^+^ T cells that infiltrated tumors from the iPS-MnO_2_@Ce6 group was significantly higher than the other groups, indicating iPS-MnO_2_@Ce6-induced T lymphocytes to accumulate in the tumor. Furthermore, ~ 27.8% more CD4^+^ helper T lymphocytes (Th) and 46.2% more CD8^+^ cytotoxic T lymphocytes (CTL) were seen in the iPS-MnO_2_@Ce6 group than in the saline control group (Fig. 6a, see also in Figs. S10 and S11), contributing directly to the enhanced killing of cancer cells. In addition, iPS-MnO_2_@Ce6-treated mice showed the highest secretion of interferon-γ (IFN-γ) and tumor necrosis factor alpha (TNF-α) cytokines, further demonstrating the strong induction of CTL-mediated cellular immunity after laser irradiation (Fig. 6b, c). Moreover, consistent with flow cytometry results, immunofluorescence experiments confirmed that iPS-MnO_2_@Ce6-mediated immunotherapy significantly increased the number of CD4^+^ and CD8^+^ T cells in tumor tissues (Fig. 6e, and Fig. S12 for other treatment groups). Increased levels of both CD8^+^ CTLs and CD4^+^ Th cells in tumors are beneficial for anti-tumor immunotherapy. However, the presence of CD4^+^CD25^+^FoxP3^+^ regulatory T cells (Tregs) in particular is known to counterbalance the immune response and acts as a major driver of immune escape in cancer [40]. Therefore, large numbers of Tregs infiltrating tumors can limit anti-tumor immune responses and affect the efficacy of immunotherapy. We found that on day 7 post-treatment, iPS-MnO_2_@Ce6-treated animals showed the lowest proportion of Tregs among all treatment groups (Fig. 6d, and Fig. S13 for flow cytometry data). These results show that iPS-MnO_2_@Ce6-mediated immunotherapy effectively improves anti-tumor response by increasing the infiltration of cytotoxic/helper T cells in tumor tissues and decreasing the proportion of Tregs that suppress anti-tumor immune responses.

We further analyzed the infiltration of macrophages (Mac), natural killer cells (NK), and bursa-dependent lymphocytes (B) of iPS-MnO_2_@Ce6-treated mice at different time points (3, 7, and 14 days) using flow cytometry. We found that the number of M1 Mac cells (CD11b^+^F4/80^+^CD206^+^) gradually increased during this time period, while that of M2 Mac cells (CD11b^+^F4/80^+^CD16/32^+^) decreased gradually (Fig. S14). Because macrophages are the main types of antigen-presenting cells for eliciting and regulating immunity, it is possible that the immune-stimulatory antigens released by iPS-MnO_2_@Ce6-treated tumor cells had induced the proliferation of M1 Mac cells and promoted their participation in antigen presentation and activation of T cells in the anti-tumor response. Over the 3, 7, and 14 day period, no significant changes were seen for B cells (CD45^+^CD^3−^CD19^+^) (Fig. 6j, and Fig. S15 for raw flow cytometry data). In contrast, the proportion of tumor-infiltrating NK cells (CD45^+^CD3^−^CD49^+^) gradually increased during the same period. Taken together, these results demonstrated that laser-mediated PDT in the presence of iPS-MnO_2_@Ce6 initiated strong anti-tumor immune responses involving multiple immune cells.

**Fig. 7:**
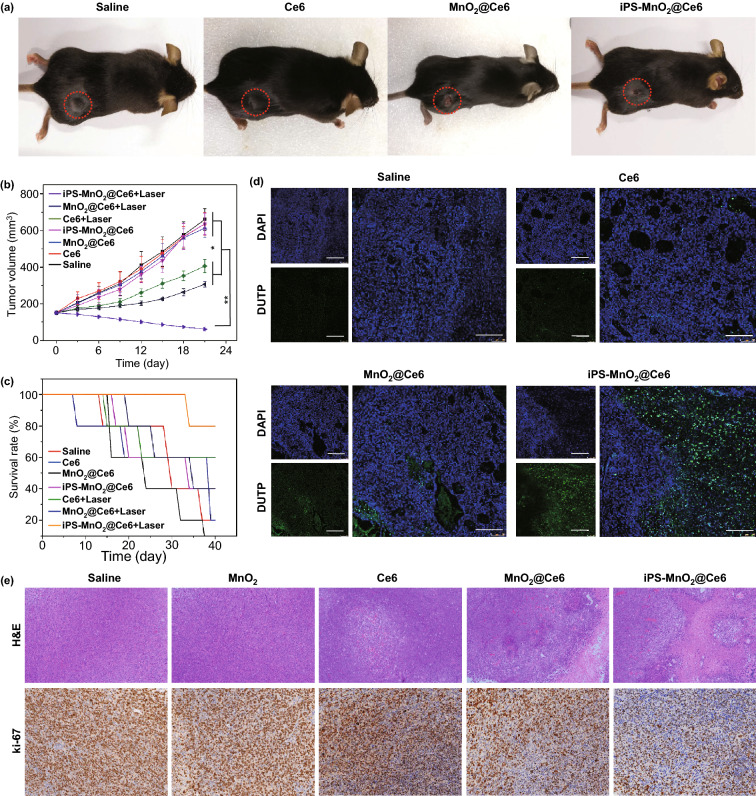
Anti-tumor activity of iPS-MnO_2_@Ce6 in vivo. **a** Representative photographs of the mice on 14th day post-combination therapy. **b** Changes of tumor volume with time increase in various groups after laser irradiation (*n* = 5 in per group). Statistical significance was calculated via two-tailed student’s *t* test. *P* value: *P < 0.01, **P < 0.001. **c** Survival curves of the mice in each group after different treatments (*n* = 5 in per group). **d** Representative TUNEL analysis of apoptosis in the tumor sections after different treatments. Scale bars are 100 μm. **e** Representative images of H&E and Ki67 stained tumor slides from the mice after different treatments. Magnification, 100 × and 200 × respectively

These anti-tumor responses manifested in LLC tumor-bearing mice treated with iPS-MnO_2_@Ce6 and laser irradiation. Of all groups, mice treated with our iPS-MnO_2_@Ce6 showed the best tumor growth inhibition (Fig. 7a, b) and highest survival rates (Fig. 7c). In contrast, animals treated with free Ce6 or MnO_2_@Ce6 and received no irradiation were unable to effectively inhibit tumor growth and had higher mortality. Additionally, TUNEL (Terminal-deoxynucleoitidyl Transferase-Mediated Nick End Labeling) assay performed on tumor sections showed that the tumors of animals treated with iPS-MnO_2_@Ce6 had large numbers of apoptotic cells (green in Fig. 7d), whereas only a small number was seen in the MnO_2_@Ce6 saline and Ce6-treated animals. Histologic analysis and of tumor tissue slides stained with hematoxylin & eosin (H&E) and Ki67 further demonstrated a decrease in malignant tumor cell density after iPS-MnO_2_@Ce6 treatment (Fig. 7e). In addition, pathological analysis of the main organs from treated tumor-bearing mice showed no obvious damage, indicating that iPS-Mn@Ce6 is biocompatible (Fig. S16). Pathological analysis also showed no tumor metastasis lesions in important organs, which indirectly suggest that prepared iPS cells loaded with MnO_2_@Ce6 could be used to fight against tumor metastasis and occurrence. When applied to other types of tumors, such as the liver and gastric cancers, similar anti-tumor responses and therapeutic effects were also observed (Figs. S18 and S19).

## Conclusions

In summary, the iPS cells loaded with MnO_2_-based nanoprobes as a novel nanoplatform are successfully developed that targets tumors, enhances the efficiency of PDT while elicits strong anti-tumor immune response. MnO_2_@Ce6 nanoparticles were loaded into mitomycin-treated iPSs to form iPS-MnO_2_@Ce6, which homed naturally to tumors. Attributed to the tumor-homing capabilities of iPS cells, the loaded MnO_2_@Ce6 nanoprobes were efficiently delivered into tumor tissues, releasing lot of oxygen by interaction between MnO_2_ and H_2_O_2_, alleviated the hypoxia in tumor, thereby facilitating an enhanced photodynamic therapy. More important, iPS cells share the similar transcriptome profiles and release the mimic antigens similar to tumor cells due to their apoptosis induced by the enhanced photodynamic therapy, recruited a stronger anti-tumor immune response, improving dendritic cells matured, enhanced the amounts of CD4+ (27.8%) and CD8+ (46.2%)T cells infiltrated into tumor tissues, decreased the amount of T regulation cells, markedly inhibited tumor growth and metastasis. In addition, we also observed that MnO_2_@Ce6 nanoprobes could activate anti-tumor immune responses in tumor-bearing animal models, the potential mechanism will be deeply investigated. Eventually, beneficial from these advantages, our iPS cells loaded with MnO_2_@Ce6 showed an intelligent and improved anti-tumor capacity based on the synergistic function of enhanced photodynamic therapy and intense tumor immunotherapy. In conclusion, human iPS cells loaded with MnO_2_@Ce6 can realize photodynamic and simultaneous enhanced immunotherapy, markedly inhibit tumor growth and occurrence and metastasis, extend lifespan of tumor-bearing animals, own great potential in clinical tumor therapeutic translation in near future.

## Electronic supplementary material

Below is the link to the electronic supplementary material.

Supplementary material 1 (PDF 2287 kb)
