# Supplementary material for: Human iPS Cells Loaded with MnO2-Based Nanoprobes for Photodynamic and Simultaneous Enhanced Immunotherapy Against Cancer
Source: Nanomicro Lett. 2020 Jun 16;12:127. doi: 10.1007/s40820-020-00452-y (PMC7770927; doi:10.1007/s40820-020-00452-y)
Supplement: Supplementary file 1 — Supplementary material 1 (PDF 2287 kb) [file 40820_2020_452_MOESM1_ESM.pdf]

Supporting Information for

Human iPS Cells Loaded with MnO_2 Based Nanoprobes for Photodynamic and Simultaneous Enhanced Immunotherapy Against Cancer

Yanlei Liu[1,#], Jingxing Yang[3,#], Bin Liu[1], Wen Cao[1], Jingpu Zhang[4], Yuming Yang[1], Lijun Ma[5], Jesus Martinez de la Fuente[1], Jie Song[1], Jian Ni[1], Chunfu Zhang[3,*], Daxiang Cui[1,2,*]

[1]Institute of Nano Biomedicine and Engineering, Key Laboratory for Thin Film and Microfabrication of Ministration of Education, Shanghai Engineering Research Centre for Intelligent Diagnosis and Treatment Instrument, Department of Instrument Science and Engineering, School of Electronic Information and Electrical Engineering, Shanghai Jiao Tong University, 800 Dongchuan Road, Shanghai 200240, People's Republic of China

[2]National Engineering Center for Nanotechnology, Shanghai 200240, People's. Republic of China

[3]Department of Nuclear Medicine, Rui Jin Hospital, School of Biomedical Engineering, Shanghai Jiao Tong University, Shanghai 200240, People's Republic of China

[4]Scientific Research Center, Shanghai Public Health Clinical Center, Fudan University, Shanghai 201508, People's Republic of China,

[5]Tongren Hospital, Shanghai Jiao Tong University School of Medicine, 1111 XianXia Road, Shanghai, 200336, People's Republic of China

[#]Yanlei Liu and Jingxing Yang contributed equally to this work

*Corresponding authors. E-mail: dxcui@sjtu.edu.cn (Daxiang Cui); cfzhang@sjtu.edu.cn (Chunfu Zhang)

Supplementary Figures

	MnO_2	MnO_2@Ce6
Zeta Potential (mv)	30 ± 3.03	10 ± 1.25

Fig. S1 Zeta potential of MnO_2 and MnO_2@Ce6

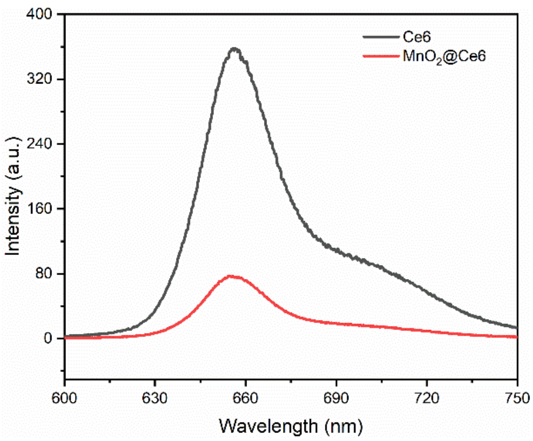

Fig. S2 Fluorescence emission spectra of Ce6 and MnO_2@Ce6

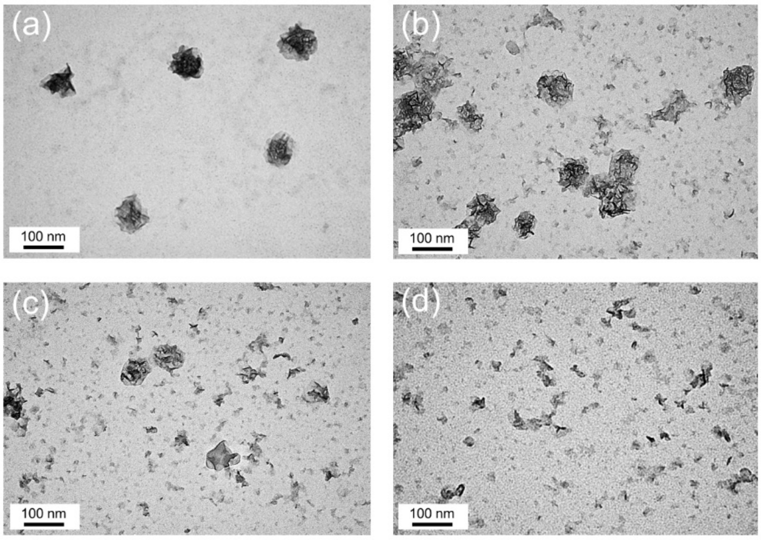

Fig. S3 TEM images of nanoprobes after incubation in PBS with different pH values for 6 h (7.4, 6.5, 5.0, 3.0)

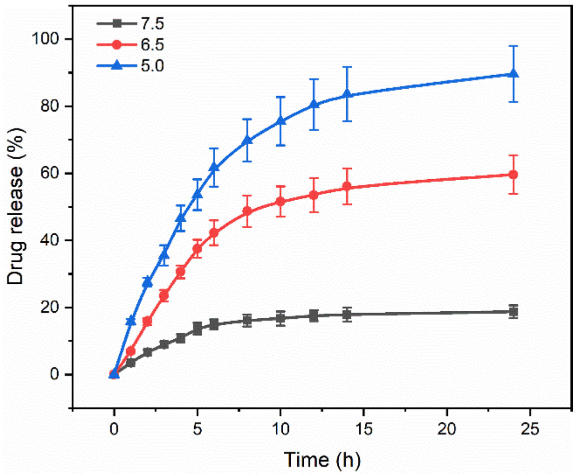

Fig. S4 Percentages of released Ce6 from MnO_2@Ce6 over time in PBS at different pH values (5.0, 6.5 and 5.5)

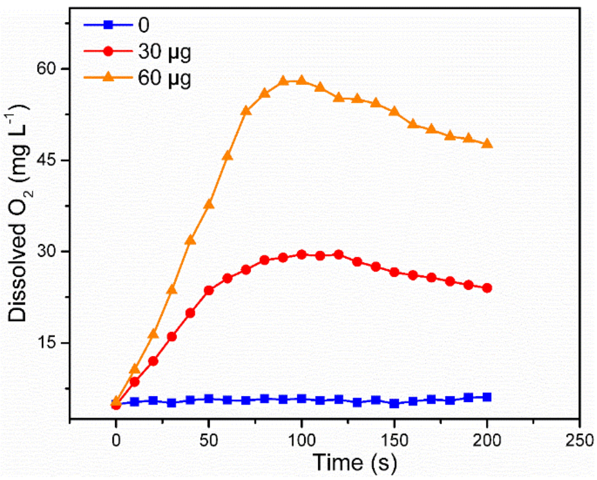

Fig. S5 O_2 generation capacity of MnO_2 in two concentrations in the presence of hydrogen peroxide (5× 10^{-3} M)

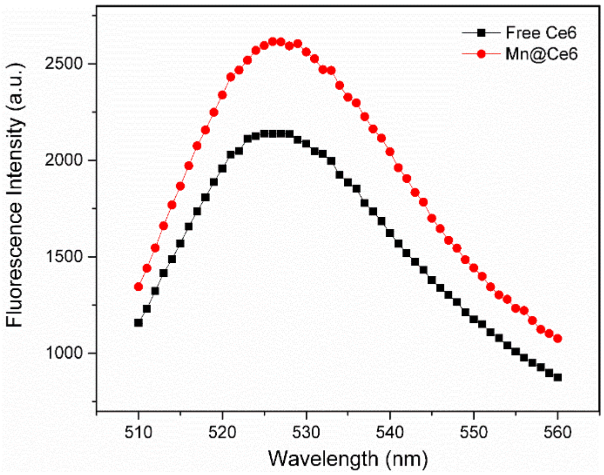

Fig. S6 SOSG fluorescence spectra of free Ce6 and MnO_2@Ce6 irradiated by laser for 3 minutes

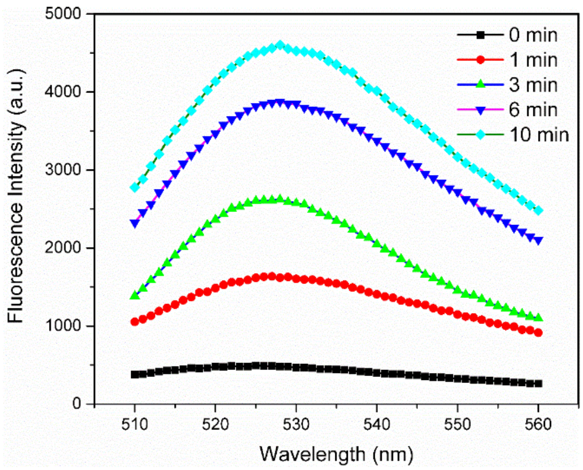

Fig. S7 SOSG fluorescence spectra of MnO_2@Ce6 irradiated by laser for different time intervals

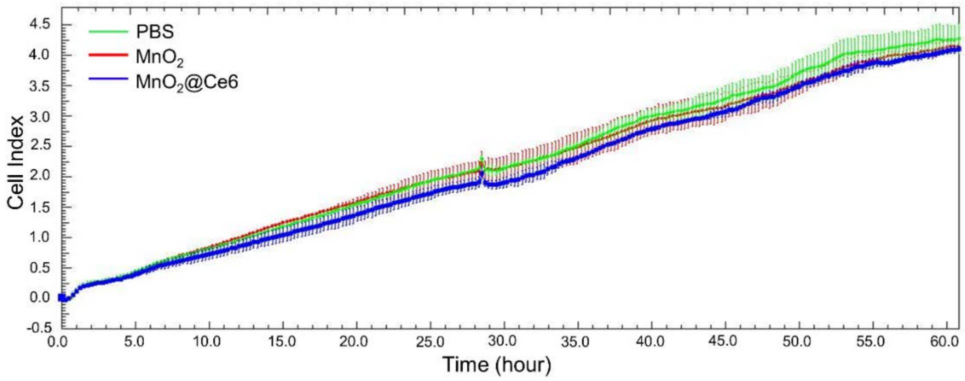

Fig. S8 Effect of nanoprobes on the proliferation of iPS cells

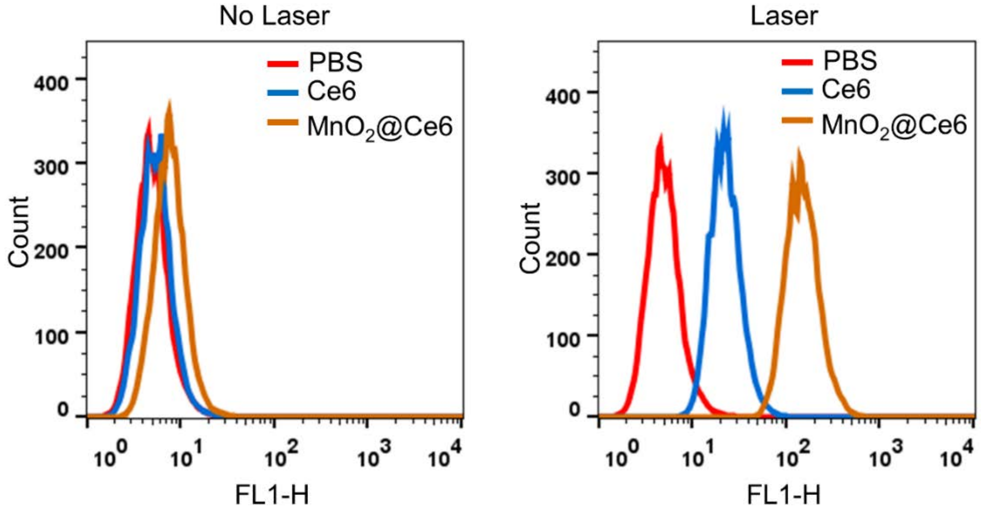

Fig. S9 Flow cytometric analysis of ROS generation in iPS cells incubated with PBS, Ce6 and Mn@Ce6 for 8 h, followed by 633 nm laser irradiation for 6 minutes (0.5 w/cm^2).

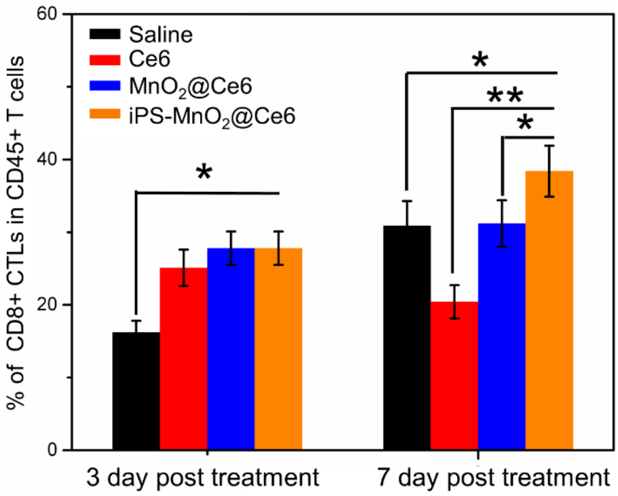

Fig. S10 Proportions of tumor-infiltrating CD8+ CTL cells in CD45+CD3+ T cells corresponding to the date in Fig. 6a

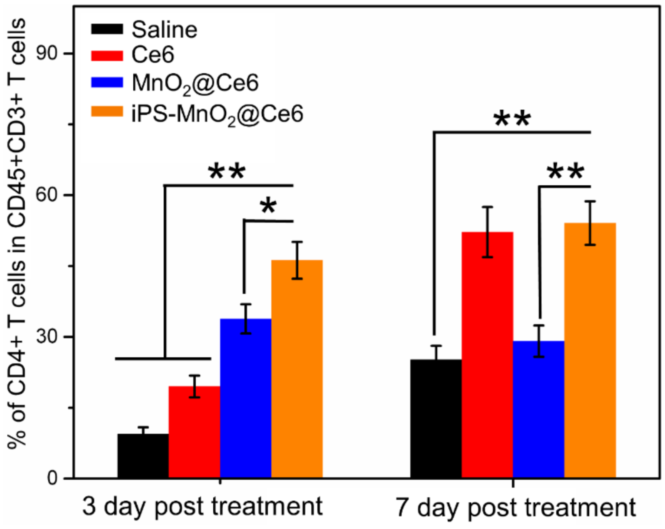

Fig. S11 Proportions of tumor-infiltrating CD4+ T cell in CD45+CD3+ T cells corresponding to the date in Fig. 6a

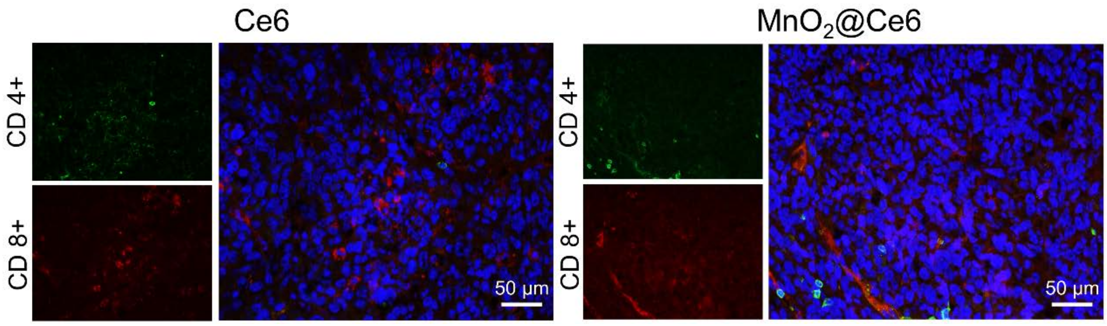

Fig. S12 Representative immunofluorescence images of tumor slides showing CD4+ and CD8+ T cells infiltrating into the tumor tissues. Green from CD4 antibody, red from CD8 antibody, and blue from DAPI

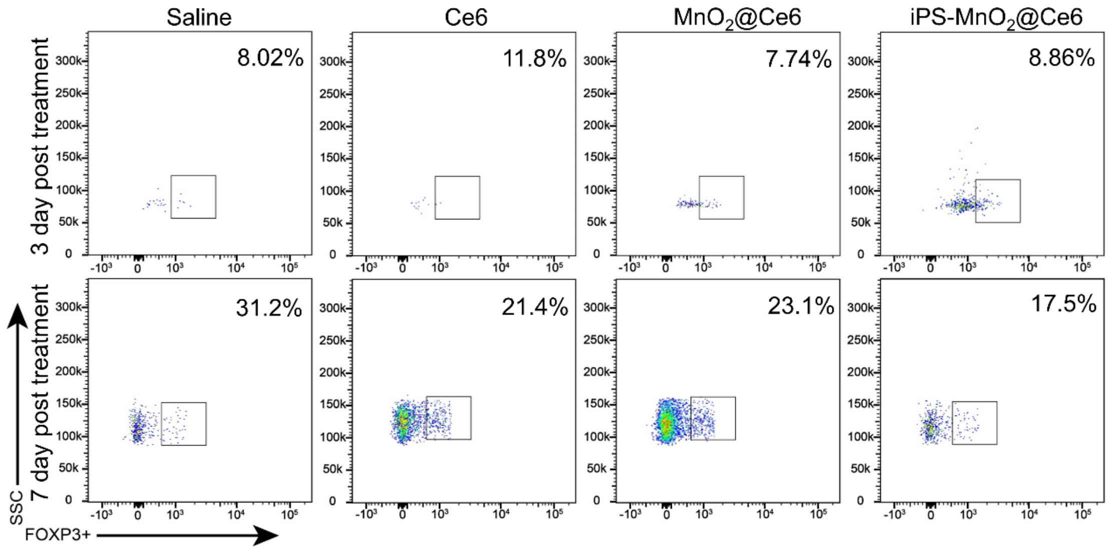

Fig. S13 Representative flow cytometry data of Treg cells infiltration in tumors

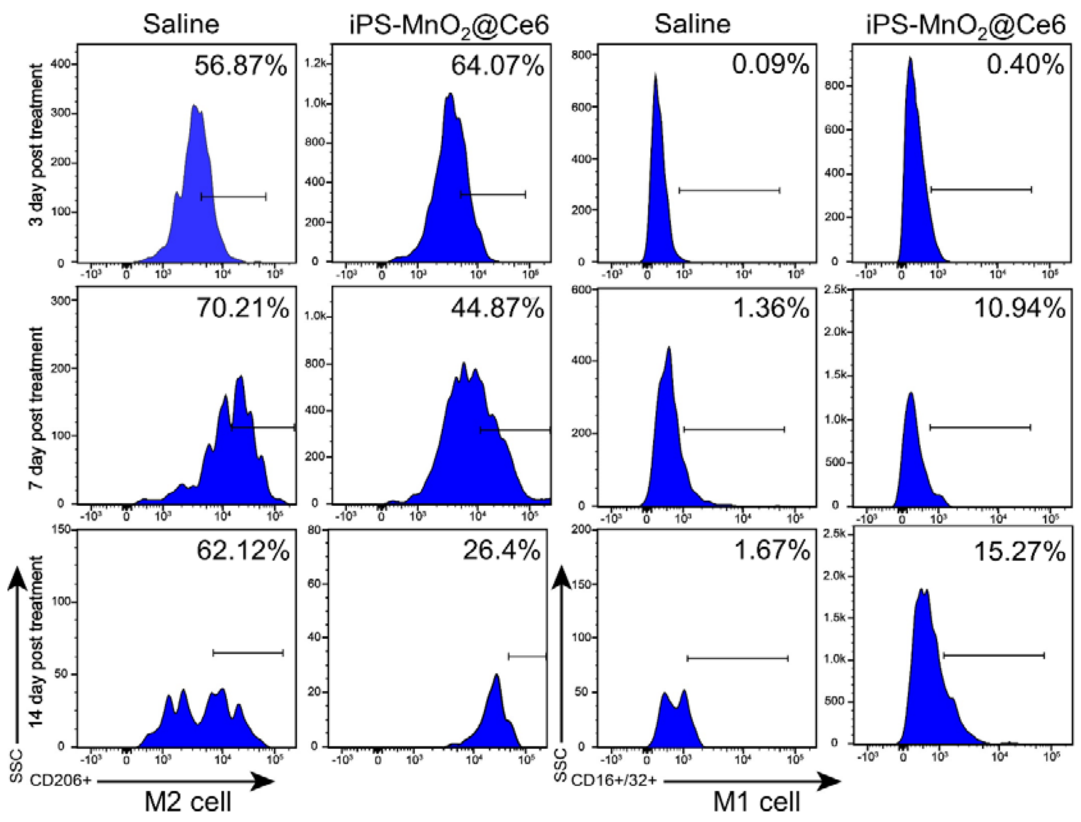

Fig. S14 Representative flow cytometry data of macrophages cells infiltration in tumors

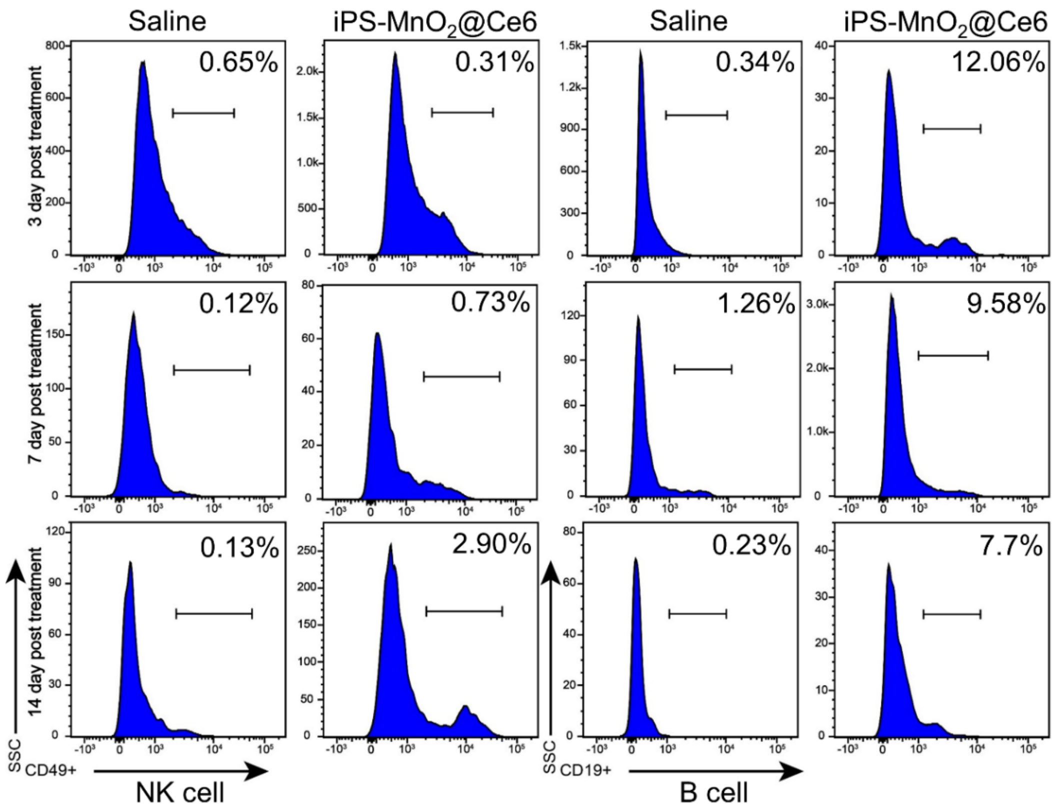

Fig. S15 Representative flow cytometry data of NK and B cells infiltration in tumors

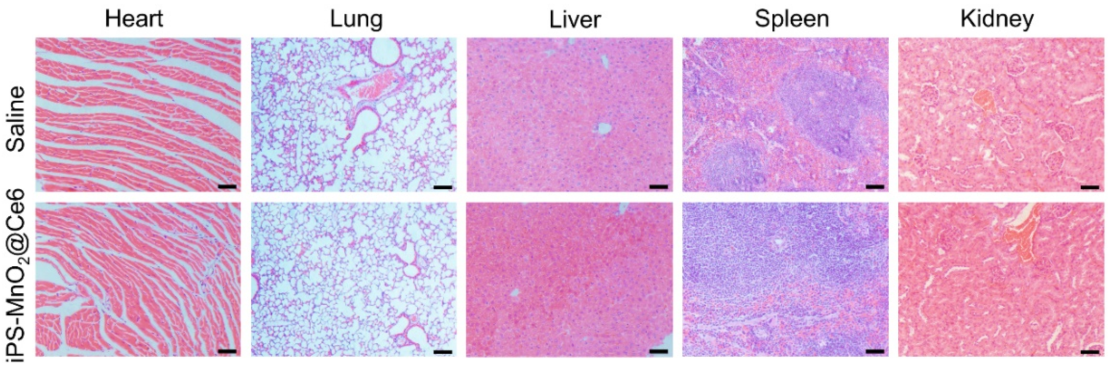

Fig. S16 Representative H&E sections of the main organs after the nanoplatform - based combination therapy. The saline group served as contrast

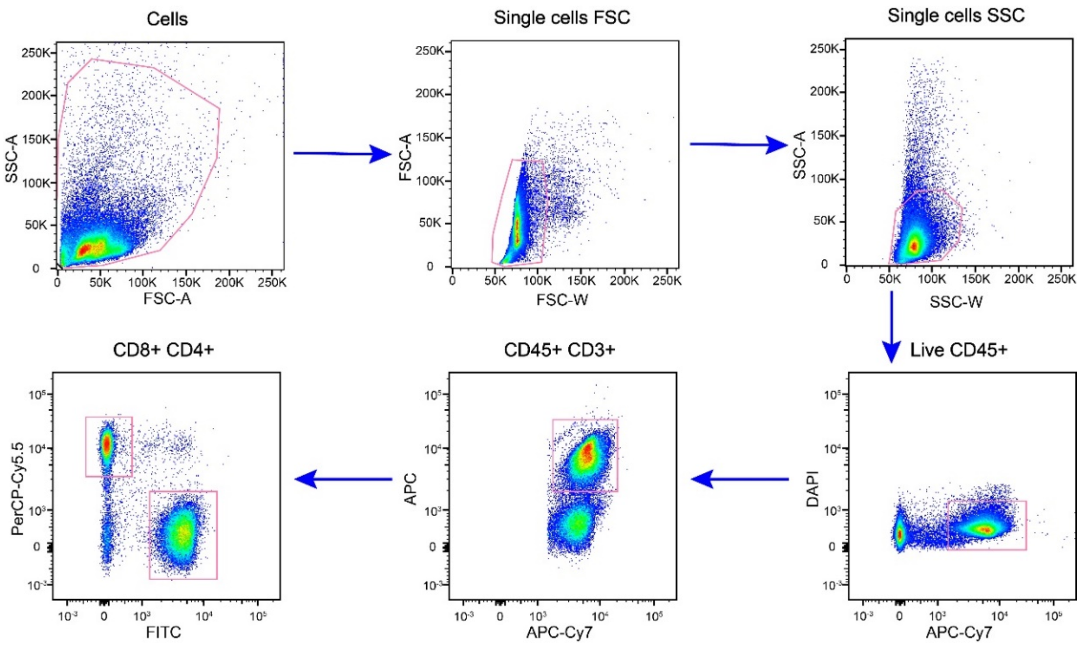

Fig. S17 Gating strategy of CD4+/CD8+ T cells in tumor sites and tumor-draining lymph nodes

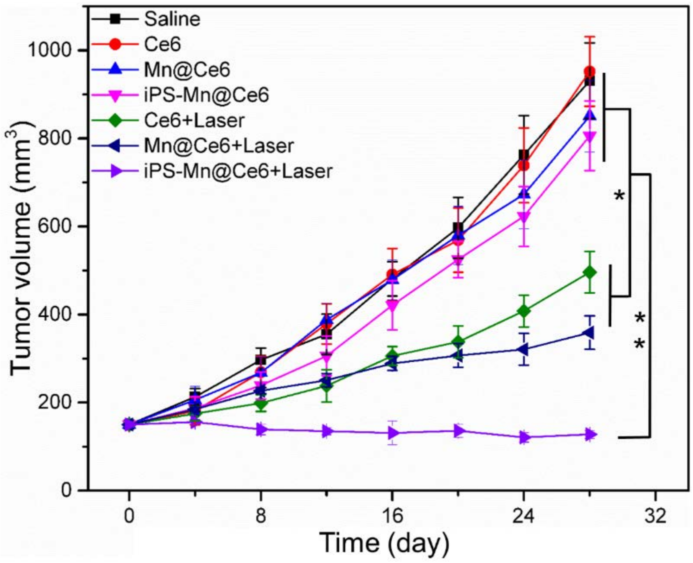

Fig. S18 Anti-tumor activity of iPS-MnO_2@Ce6 in vivo on the liver cancer model. Changes of tumor volume with time increase in various groups after laser irradiation (n=5 in per group). Statistical significance was calculated via two-tailed Student's -test. *P* value: **P*<0.01, ** *P*<0.001. (b) Survival curves of the mice in each group after different treatments (n=5 in per group)

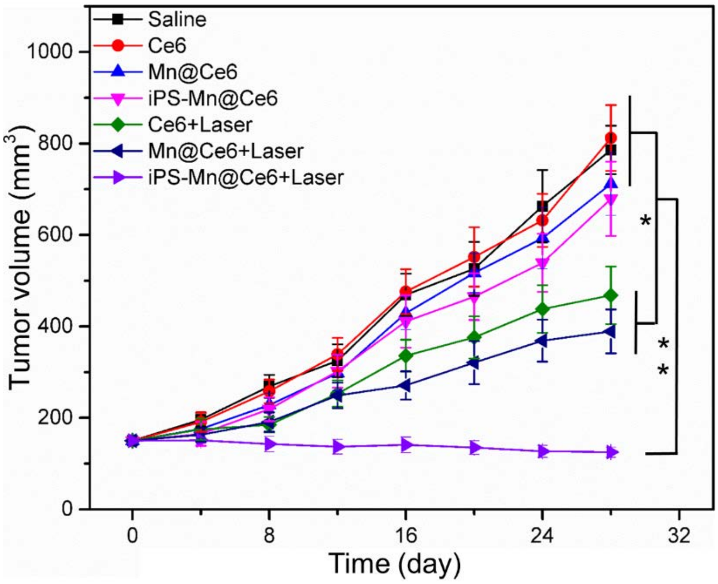

Fig. S19 Anti-tumor activity of iPS-Mn@Ce6 in vivo on the gastric cancer model. (a) Changes of tumor volume with time increase in various groups after laser irradiation (n=5 in per group). Statistical significance was calculated via two-tailed Student's -test. *P* value: **P*<0.01, ** *P*<0.001. (b) Survival curves of the mice in each group after different treatments (n=5 in per group)
